# CYP2A6 and GABRA2 Gene Polymorphisms are Associated With Dexmedetomidine Drug Response

**DOI:** 10.3389/fphar.2022.943200

**Published:** 2022-07-07

**Authors:** Chao Fang, Wen Ouyang, Youjie Zeng, Qi Pei, Yuhao Xia, Siwan Luo, Minghua Chen

**Affiliations:** ^1^ Department of Anesthesiology, Third Xiangya Hospital, Central South University, Changsha, China; ^2^ Hunan Cancer Hospital, The Affiliated Cancer Hospital of Xiangya School of Medicine, Central South University, Changsha, China; ^3^ Department of Pharmacy, Third Xiangya Hospital, Central South University, Changsha, China

**Keywords:** dexmedetomidine, pharmacogenomics, pharmacokinetic, pharmacodynamics, GABA A receptor, *CYP2A6*

## Abstract

**Background:** Dexmedetomidine is a commonly used clinical sedative; however, the drug response varies among individuals. Thus, the purpose of this study was to explore the association between dexmedetomidine response and gene polymorphisms related to drug-metabolizing enzymes and drug response (*CYP2A6*, *UGT2B10*, *UGT1A4*, *ADRA2A*, *ADRA2B*, *ADRA2C*, *GABRA1*, *GABRB2*, and *GLRA1*).

**Methods:** This study was a prospective cohort study. A total of 194 female patients aged 18–60 years, American Society of Anesthesiologists (ASA) score I-II, who underwent laparoscopy at the Third Xiangya Hospital of Central South University, were included. The sedative effect was assessed every 2 min using the Ramsay score, and the patient’s heart rate decrease within 20 min was recorded. Peripheral blood was collected from each participant to identify genetic variants in the candidate genes of metabolic and drug effects using the Sequenom MassARRAY^®^ platform. Furthermore, additional peripheral blood samples were collected from the first 99 participants at multiple time points after dexmedetomidine infusion to perform dexmedetomidine pharmacokinetic analysis by Phoenix^®^ WinNonlin 7.0 software.

**Results:** Carriers of the minor allele (C) of *CYP2A6* rs28399433 had lower metabolic enzyme efficiency and higher plasma concentrations of dexmedetomidine. In addition, the participants were divided into dexmedetomidine sensitive or dexmedetomidine tolerant groups based on whether they had a Ramsay score of at least four within 20 min, and *CYP2A6* rs28399433 was identified to have a significant influence on the dexmedetomidine sedation sensitivity by logistic regression with Plink software [*p* = 0.003, OR (95% CI): 0.27 (0.11–0.65)]. C allele carriers were more sensitive to the sedative effects of dexmedetomidine than A allele carriers. *GABRA2* rs279847 polymorphism was significantly associated with the degree of the heart rate decrease. In particular, individuals with the GG genotype had a 4-fold higher risk of heart rate abnormality than carriers of the T allele (OR = 4.32, 95% CI: 1.96–9.50, *p* = 0.00027).

**Conclusion:** *CYP2A6* rs28399433 polymorphism affects the metabolic rate of dexmedetomidine and is associated with susceptibility to the sedative effects of dexmedetomidine; *GABRA2* rs279847 polymorphism is significantly associated with the degree of the heart rate decrease.

## Introduction

Dexmedetomidine is a highly selective adrenergic agonist that inhibits noradrenaline release, producing sedation, analgesic, and anxiolytic effects (Lee, 2019). It has been widely used as an adjunct to general anesthesia or for sedation in the intensive care unit (ICU) due to its advantages of intraoperative and postoperative analgesia, sedation without respiratory depression, and low incidence of postoperative nausea and vomiting (Liu et al., 2021).

The mechanism of the sedative and analgesic effects of dexmedetomidine is not fully understood. The majority of studies suggest that dexmedetomidine inhibits the release of norepinephrine by activating alpha2-adrenergic receptors in the locus coeruleus, thereby blocking the transmission of pain signals and inducing sedation and analgesia (Correa-Sales et al., 1992). The metabolism of dexmedetomidine is mainly achieved through hepatic biotransformation, including CYP2A6 hydroxylation metabolism and glucuronidation by UGT2B10 and UGT1A4 (Cortinez et al., 2015). The primary metabolites of dexmedetomidine are the N-glucuronide lipid isomers (G-Dex-1, G-Dex-2) and the O-glucuronide lipid hydroxylation N-methyl-dexmedetomidine H1 and imidazole oxidation product H3, which are 100-fold less potent in the alpha-2 receptor and are considered to be inactive (Gertler et al., 2001).

The sedative effect of dexmedetomidine varies between individuals. A 6-month prospective observational study revealed that approximately half of the participants in the intensive care unit did not achieve satisfactory sedative effect after intravenous infusion of a predetermined dose of dexmedetomidine (Smithburger et al., 2014). The most common side effects are hemodynamic variations such as initial hypertension, bradycardia, and hypotension, which correlate to the plasma drug concentration (Colin et al., 2017). Thus, it is necessary to individualize the dosing to achieve a more satisfactory sedative effect and reduce cardiovascular side effects.

The genetic factor is one of the contributors to the variations in drug efficacy and adverse effect severity between individuals given the same drug dose (Bachtiar et al., 2019). Single nucleotide polymorphism (SNP) is the most common heritable genetic variation (Chaudhary et al., 2015). There exist numerous SNPs in the genes encoding metabolizing enzymes, transporter proteins, and receptor proteins (Ahmed et al., 2016; Cacabelos et al., 2019). However, not every SNP has a clinical impact. Therefore, it is essential to identify SNPs that influence pharmacokinetics and pharmacodynamics.

Studies on the influence of genetic factors on the dexmedetomidine drug effect are currently scarce. Several studies have suggested that *CYP2A6* gene polymorphisms seem not to influence the pharmacokinetics of dexmedetomidine (Kohli et al., 2012; Wang et al., 2018). However, there may be limitations with the relatively poor sample sizes of these studies. By the way, the complex genetic architecture of *CYP2A6* coupled with its significant homology with other *CYP2A* genes (*CYP2A7* and *CYP2A13*) makes genotyping challenging (Ding and Kaminsky, 2003; Di et al., 2009). The effect of alpha-2 adrenergic receptor gene polymorphisms on the cardiovascular response after dexmedetomidine infusion is currently inconclusive (Talke et al., 2005a; Talke et al., 2005b; Muszkat et al., 2005; Yagar et al., 2011; Zhu et al., 2019). GABA is the main inhibitory neurotransmitter of the central nervous system (Fatemi et al., 2013). Philbin et al. reported that alpha2-receptor agonist clonidine diminishes GABAergic neurotransmission to cardiac vagal neurons in the nucleus ambiguous, which result in decrease in heart rate and blood pressure (Philbin et al., 2010). Nevertheless, few studies have reported whether SNPs of GABA_A_ receptor genes affect the heart rate decrease after dexmedetomidine infusion. Overall, there are still multiple SNPs in dexmedetomidine metabolizing enzymes and cardiovascular response-related genes that remain unexplored.

The aim of our study is to explore the association between dexmedetomidine response and gene polymorphisms related to drug-metabolizing enzymes and drug response (*CYP2A6*, *UGT2B10*, *UGT1A4*, *ADRA2A*, *ADRA2B*, *ADRA2C*, *GABRA1*, *GABRB2*, and *GLRA1*).

## Materials and Methods

### Study Participants

From August 2018 to May 2019, 194 Chinese female patients who underwent laparoscopy under general anesthesia at The Third Xiangya Hospital of Central South University were recruited. Inclusion criteria: 1) Female patients aged 18–60 years undergoing laparoscopic with ASA I-II [ASA I: normal healthy patients; ASA II: patients with mild systemic disease (Doyle et al., 2022)] and a body mass index (BMI) of 18–28 kg/m^2^; 2) participants must have given informed consent to the study and signed a written informed consent form; 3) participants were able to communicate with the investigator and completed the study in accordance with the study regulations. Exclusion criteria: 1) abnormal liver or renal function (elevated AST or ALT levels [≥ 2 times]; elevated Scr levels [≥ 1.2 times]); 2) severe hypotension or complete atrioventricular block; 3) endocrine disorders (e.g., abnormal thyroid function); 4) drug allergy history; 5) administration of an alpha agonist within two weeks such as colistin; 6) Medications that influence hepatic metabolism within 30 days previous to our study (e.g., barbiturates, carbamazepine, phenytoin, glucocorticoids, omeprazole, SSRI antidepressants, cimetidine, diltiazem, macrolides, nitroimidazoles, verapamil, fluoroquinolones, antihistamines); 7) Participation in blood donation (>400 ml) or receiving blood transfusion within three months previous to surgery; 8) Soft drug (e.g., marijuana) abuse within three months previous to surgery or hard drug (e.g., cocaine, phencyclidine, etc.) abuse within one year previous to surgery; 9) pregnancy or oral contraceptives usage 30 days before surgery; 10) long-term special diet and alcohol consumption; 11) Patients refuse to participate. The study has been registered with the China Clinical Trials Registry (registration number: ChiCTR1800017474; https://www.chictr.org.cn/index.aspx). The study was approved by the ethics committee of The Third Xiangya Hospital of Central South University (registration number: R18017). The study was conducted following the Declaration of Helsinki, and each patient signed a written informed consent.

### Treatments

All patients were given no preoperative medication. Non-invasive blood pressure (NIBP), oxygen saturation (SpO_2_), electrocardiogram (ECG), and bispectral index (BIS) were routinely monitored. Oxygen was administered by nasal cannula (2L/min). Dexmedetomidine was continuous intravenous infused at 1 μg/kg for 10 min before the induction period of general anesthesia.

Ten minutes after the end of dexmedetomidine infusion, induction of anesthesia was performed in the usual way (propofol 1.5–2.5 mg/kg, midazolam 0.02–0.05 mg/kg, sufentanil 0.2–0.5 μg/kg, and cis-atracurium 0.2–0.4 mg/kg). After tracheal intubation, appropriate ventilator parameters were set to maintain PaCO_2_ 35–45 mmHg. General anesthesia was maintained by continuous pumping of propofol and remifentanil, and BIS was maintained 45–60 during the anesthesia period.

During the study, atropine 0.3–0.5 mg was given when the patient’s heart rate was below 50 per minute, and ephedrine 5 mg was administered when the mean arterial pressure (MAP) was 30% below the basal value or when the NIBP was below 90/60 mmHg.

### Peripheral Blood Sampling

This study included 194 participants, of which pharmacokinetic studies were performed on 99 participants, and pharmacodynamic studies were performed on all participants. All participants had 5 ml of peripheral blood sampled preoperatively for DNA isolation and genetic testing. All blood samples were preserved in EDTA-2K anticoagulation tubes. The peripheral blood samples were stored at −20°C until DNA was isolated.

For the first 99 participants, 5 ml of peripheral blood samples were additionally collected at 2, 5, and 10 min after dexmedetomidine administration, and 5, 10, 20, 30, and 45 min, 1, 1.5, 2, and 8 h after the ending of dexmedetomidine infusion to determine dexmedetomidine pharmacokinetics. These 99 blood samples were centrifuged at 3500 rpm, and the plasma was extracted to determine the pharmacokinetics of dexmedetomidine. The plasma samples were stored at −80°C until dexmedetomidine concentration was determined.

### Determination of the Concentration of Dexmedetomidine in Plasma by HPLC Analytical Technique

Plasma drug concentration monitoring by HPLC-MS/MS-based method has been reported in the previous study (Chang et al., 2020). Gradient elution was performed on a CHIRALPAK^®^ AGP 5 μm, 2.1 × 150 mm column with dexmedetomidine -d4 as an internal standard. The ion source is an electrospray ion source (ESI) with positive ion multiple reaction monitoring (MRM) mode detections. The calibrated range of the method was 1.0–2000 ng/ml for all analytes. The intra- and inter-day precisions (CV, %) were <15%, and the accuracies (%) were within the range of 85.0–115.0%.

### Identification of the Clinical Pharmacological Effects of Dexmedetomidine

The Ramsay scale was used to assess the sedative effect every 2 min for 20 min after administration of dexmedetomidine. Ramsay scale scoring criteria: 1 = unquiet and irritable; 2 = quiet and cooperative; 3 = respond to command only; 4 = wakeable sleep states; 5 = sluggish response to call; 6 = deep sleep with no response to call (Mondello et al., 2002). In this study, patients with a Ramsy score of at least four within 20 min were considered sensitive to the sedative effects of dexmedetomidine. In addition, a heart rate (HR) of less than 50 beats per minute is considered an HR abnormality.

### Screening of TagSNPs in Candidate Genes

Genes involved in dexmedetomidine metabolism, sedative effects, and cardiovascular responses were screened through literature review. These genotyping data were then downloaded from the Ensemble database (http://asia.ensembl.org/index.html) and imported into HaploView 4.2 software. This software is available to identify tagSNPs, which are representative SNPs for genomic regions with high linkage disequilibrium and are used to characterize the variation information of the whole gene (Barrett et al., 2005). The screening selection criteria were set to a minimum allele frequency (MAF) > 0.05 and a linkage coefficient *r* ^2^ ≥ 0.8. Twenty-two tagSNPs were finally screened (Table 1).

**TABLE 1:** Genotyping results of the tagSNPs.

Gene	SNP	Allele	Call rate (%)	MAF (%)	HWE *p* Value
*CYP2A6*	rs143731390	T > A	98.97	10.82	0.14
*CYP2A6*	rs28399433	A > C	99.48	28.06	0.48
*CYP2A6*	rs28399481	C > T	NA	NA	NA
*CYP2A6*	rs5031016	A > G	NA	NA	NA
*UGT2B10*	rs115455627	C > T	97.94	22.68	0.11
*UGT2B10*	rs11940320	G > A	100	10.61	1
*UGT1A4*	rs12468356	A > G	100	17.17	0.65
*UGT1A4*	rs8330	C > G	100	9.60	0.70
*UGT1A4*	rs2008584	A > G	98.45	24.74	<0.05^a^
*ADRA2A*	rs2484516	C > G	98.45	8.639	1
*ADRA2A*	rs3750625	C > A	98.45	20.94	<0.05^a^
*ADRA2B*	rs4907299	T > G	100	47.16	0.66
*ADRA2B*	rs2229169	G > T	100	45.88	0.77
*ADRA2C*	rs7434444	G > C	100	26.8	<0.05^a^
*ADRA2C*	rs11269124	dupGC(T)3	99.48	32.64	0.33
		(C)3AGAG			
		A(C)3(G)4A			
*GABRA1*	rs11576001	A > G	100	46.13	0.56
*GABRA1*	rs77445936	T > C	100	20.36	0.82
*GABRA2*	rs279847	T > G	100	46.91	0.15
*GABRA2*	rs10433685	G > C	100	36.86	0.06
*GABRB2*	rs10214094	A > G	99.48	8.29	0.37
*GLRA1*	rs4076138	T > C	100	28.35	1
*GLRA1*	rs4958283	A > G	98.97	39.43	0.45

a p < 0.05, indicating that UGT1A4 rs2008584, ADRA2A rs3750625, and ADRA2C rs7434444 did not follow the Hardy-Weinberg equilibrium.

CYP2A6, cytochrome P450 family 2 subfamily A member 6; UGT2B10, UDP glucuronosyltransferase 2 family, polypeptide B10; UGT1A4, UDP glucuronosyltransferase 1 family, polypeptide A4; ADRA2A, adrenoceptor alpha 2A; ADRA2B, adrenoceptor alpha 2B; ADRA2C, adrenoceptor alpha 2C; GABRA1, gamma-aminobutyric acid A receptor, alpha 1; GABRA2, gamma-aminobutyric acid A receptor, alpha 2; GABRB2, gamma-aminobutyric acid A receptor, beta 2; GLRA1, glycine receptor alpha 1; SNP, single nucleotide polymorphism; MAF, minor allele frequency (was calculated with our cohort); HWE, Hardy-Weinberg equilibrium.

### DNA Isolation and SNPs Genotyping

Genomic DNA was isolated from the peripheral blood using the Magen HiPure Blood DNA Mini Kit. UV spectrophotometer and agarose gel electrophoresis were used to examine the concentration and quality of DNA. The isolated DNA samples were stored at −20°C for subsequent genotyping.

The Sequenom MassARRAY^®^ platform was used for genotyping. Specific amplification primers were designed based on the SNP information (Supplementary Table S1). The fragment of DNA containing the SNP was then amplified. The residual dNTPs were neutralized by shrimp alkaline phosphatase, and single base extensions were then performed using site-specific primers. Extension products were spotted on the chip after resin purification. SpectroCHIP was then compactly analyzed by the MassARRAY analyzer. The genotypes of target sites were interpreted based on the mass spectrum peaks.

### Statistical Analysis

The data were analyzed using SPSS software (version 22.0, SPSS, Chicago, United States) and PLINK 1.07 (http://pngu.mgh.harvard.edu/purcell/plink/). The pharmacokinetic parameters were carried out by non-compartmental analyses (NCA) in Phoenix^®^ WinNonlin 7.0 software. The chi-square test (χ2-test) in PLINK software was applied to perform the Hardy-Weinberg equilibrium analysis. The measurement data were described using the mean ± SD, and the normality test was performed by Kolmogorov-Smirnov test in SPSS. Comparisons between two groups were performed using the independent samples *t*-test, and comparisons of differences between three groups were performed using analysis of variance (ANOVA). For non-normally distributed data, the Mann-Whitney U test was used for comparison between two groups, and the Kruskal-Wallis H was used for comparison between three groups. Logistic regression models in PLINK software were used to analyze the relationship between each genotype and sedation scores in the study population and the relationship between each genotype and the patient’s heart rate. Bonferroni correction was used for multiple testing. Power test calculations were carried out using G*Power (Version 3.1.9.4, Germany).

## Results

### Genotyping Results

In our study, 22 SNPs in 194 participants were genotyped. The genotype distributions are shown in Table 1. No genotyping results were available for *CYP2A6* rs28399481 and rs5031016. The call rates for the remaining SNPs were >95%. *UGT1A4* rs2008584, *ADRA2A* rs3750625 and *ADRA2C* rs7434444 did not conform to the Hardy Weinberg equilibrium (*p* < 0.05). The remaining 17 SNPs were included for subsequent analysis.

### Effect of Gene Polymorphisms on Pharmacokinetic Parameters of Dexmedetomidine

The impact of seven metabolic enzyme gene SNPs on the pharmacokinetic parameters of dexmedetomidine in the former 99 participants was analyzed. Drug concentration-time profiles in the 99 participants were plotted by the xy-plot function of Phoenix^®^ WinNonlin 7.0. The pharmacokinetic parameters were carried out by non-compartmental analyses (NCA) in Phoenix^®^ WinNonlin 7.0 software. The results indicated that *CYP2A6* rs28399433 had a significant effect on Cl_obs and had a marginal statistical effect on C_max_ and AUC_INF__obs. After performing weight correction, *CYP2A6* rs28399433 maintained a significant effect on Cl_obs (test power = 0.77). In addition, moderately statistically effects on AUC_last_ and AUC_INF__obs were identified, and no influence was detected on C_max_ (Table 2). The pharmacokinetic parameters for each genotype of the remaining SNPs are shown in Supplementary Table S2. These results indicated that carriers of the minor allele (C) of *CYP2A6* rs28399433 have lower metabolic enzyme efficiency and higher plasma concentrations of dexmedetomidine.

**TABLE 2:** Effects of *CYP2A6* rs28399433 on pharmacokinetic parameters of dexmedetomidine.

*CYP2A6* rs28399433	AA (51)	AC (41)	CC(7)	*p* Value
HL_Lambda_z (h)	2.55 ± 0.73	2.41 ± 0.89	2.96 ± 0.86	0.09
C_max_ (ng/L)	1912.82 ± 425.52	2225.48 ± 859.28	2451.77 ± 772.75	0.047^a^
AUC_last_ (h*ng/L)	1353.22 ± 385.76	1486.56 ± 459.38	1518.98 ± 692.19	0.051
AUC_INF__obs (h*ng/L)	1540.94 ± 412.17	1680.44 ± 540.61	1801.72 ± 1071.77	0.044^a^
Vz_obs (L)	130.95 ± 39.52	115.37 ± 31.72	135.38 ± 66.99	0.165
Cl_obs (L/h)	37.02 ± 9.93	36.64 ± 14.69	32.52 ± 6.80	0.031^a^
C_max_/kg (ng/L/kg)	35.75 ± 7.26	40.48 ± 16.13	44.63 ± 13.61	0.089
AUC_last_/kg (h*ng/L/kg)	25.44 ± 7.30	27.13 ± 8.67	34.52 ± 14.52	0.032^b^
AUC_INF__obs/kg (h*ng/L/kg)	28.92 ± 7.82	30.66 ± 10.04	32.10 ± 18.62	0.049^b^
Vz_obs/kg (L/kg)	2.49 ± 0.98	2.09 ± 0.51	2.34 ± 1.12	0.472
Cl_obs/kg (L/h/kg)	0.70 ± 0.21	0.67 ± 0.29	0.48 ± 0.12	0.027^b^

a p < 0.05, indicating that CYP2A6 rs28399433 had a significant effect on Cl_obs, and had a marginal statistical effect on C_max_ and AUC_INF__obs.

b p < 0.05, after performing weight correction, CYP2A6 rs28399433 maintained a significant effect on Cl_obs. In addition, moderately statistically effects on AUC_last_ and AUC_INF__obs were identified.

HL_Lambda_z, elimination half-life; C_max_, maximum concentration; AUC_last_, area under curve for the period from the start of the infusion time to the last blood sample collection point; AUC_INF_obs_, area under curve for the period from the start of the infusion time to infinity; Vz_obs, apparent volume of distribution; Cl_obs, clearance.

### Effect of Gene Polymorphisms on Clinical Pharmacodynamics of Dexmedetomidine

The effect of 17 SNPs on the sedative effect and the degree of the heart rate decrease after dexmedetomidine infusion in 194 participants was analyzed. The participants were divided into dexmedetomidine sensitive or dexmedetomidine tolerant groups based on whether they had a Ramsay score of at least four within 20 min *CYP2A6* rs28399433 was identified to be associated with the susceptibility to the sedative effect of dexmedetomidine by chi-square (χ2) test (Table 3; *p* = 0.001, OR [95% CI]: 0.23 [0.09–0.60], test power = 0.82). This SNP remained significant influence by logistic regression with Plink software (*p* = 0.003, OR [95% CI]: 0.27 [0.11–0.65]). After correcting for the covariates (age and weight), the *p*-value remained statistically significant (*p* = 0.005, OR [95% CI]: 0.25 [(0.10–0.65]). The above results are shown in Table 3. We further analyzed the relationship between pharmacokinetics and sedative effects in the first 99 participants and identified a significant correlation between Cmax and sedative effects (Table 4, test power = 0.83). The results of SNPs with no statistically significant influence on the sedative effect are shown in Supplementary Table S3. In summary, our results indicated that C allele carriers were more sensitive to the sedative effects of dexmedetomidine than A allele carriers.

**TABLE 3:** Association of *CYP2A6* rs28399433 with the sedative effect of dexmedetomidine.

Polymorphism	Genotype	Ramsay<4	Ramsay≥4	*P*_1_	OR_1_ (95%CI)	*P*_2_	OR_2_ (95%CI)	*P*_*ad*just_	OR_*ad*just_ (95%CI)
*CYP2A6* rs28399433	AA	21	80	**0.001** ^a^	0.23 (0.09–0.60)	**0.003** ^b^	0.27 (0.11–0.65)	**0.005** ^c^	0.25 (0.10–0.65)
	AC	5	69						
	CC	0	18						

a P_1_ < 0.05, following the Chi-square test, statistically significant differences in sedative effects were identified between participants with AA, AC and CC genotypes (CYP2A6 rs28399433).

b P_2_ < 0.05, by logistic regression analysis, CYP2A6 rs28399433 was identified to significantly affect the sedative effect after dexmedetomidine infusion.

c P_adjust_ < 0.05, after correction for age and body weight, CYP2A6 rs28399433 remained a significant influence on the sedative effect after dexmedetomidine infusion.

**TABLE 4:** Relationship between pharmacokinetic parameters and sedative effects of dexmedetomidine.

	Ramsay≥4	Ramsay<4	*p* Value
HL_Lambda_z	2.56 ± 0.89	2.37 ± 0.46	0.497
C_max_ (ng/L)	2148.60 ± 684.72	1686.18 ± 495.42	**0.034** ^a^
AUC_last_ (h*ng/L)	1486.57 ± 491.38	1281.91 ± 351.88	0.186
AUC_INF__obs (h*ng/L)	1732.14 ± 637.12	1370.64 ± 373.14	0.070

a p < 0.05, indicating a significant correlation between C_max_ and sedative effects.

**FIGURE 1:**
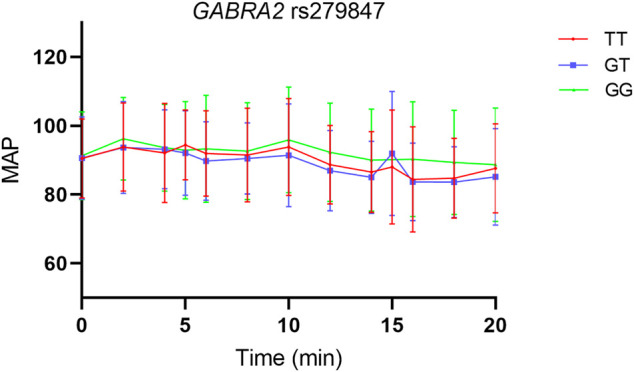
Trends in MAP within 20 min in Patients with Different Genotypes of *GABRA2* rs279847.

Abnormal heart rate due to dexmedetomidine, which incidence was 19.69%, was defined as a heart rate less than 50 per min at least once within 20 min. The results suggest that the GABRA2 rs279847 polymorphism is significantly associated with the heart rate decrease. In particular, individuals with the GG genotype had a 4-fold higher risk of heart rate abnormality than carriers of the T allele (OR = 4.32, 95% CI: 1.96–9.50, *p* = 0.00027, test power = 0.91) (Table 5). To exclude the effect of blood pressure on heart rate, we plotted the trend of mean arterial pressure change in individuals with different genotypes of GABAA2 rs279847 within 20 min (Figure 1). There was no significant difference in MAP between different genotypes. The association of other SNPs with heart rate abnormality is shown in Supplementary Table S4.

**TABLE 5:** Association of *GABRA2* rs279847 with abnormal heart rate due to dexmedetomidine.

SNP	Genotype	HR < 50		Models	OR (95%CI)	*p* Value	*P*-Adjust^a^
		Y	N				
*GABRA2* (rs279847)	TT	7	53				
	TG	13	72				
	GG	18	30	Additive^b^	2.47 (1.45–4.21)	0.00085	0.014
				Dominant^c^	2.48 (1.01–6.11)	0.049	0.78
				Recessive^d^	4.32 (1.96–9.50)	0.00027	0.0044

a P-adjust with Bonferroni.

b Additive = TT vs. TG vs. GG.

c Dominant = TT vs. GT + GG.

d Recessive = TT + TG vs. GG.

## Discussion

Our study indicates that *CYP2A6* rs28399433 affects the metabolism rate of dexmedetomidine and is associated with sensitivity to the sedative effects of dexmedetomidine. In addition, the *GABRA2* rs279847 polymorphism is significantly associated with the degree of the heart rate decrease.

Pharmacogenomic studies have shown that genetic variation is the leading cause of inter-individual differences in drug metabolism (Waring, 2020). CYP2A6 is the main metabolic enzyme mediating the hydroxylation of dexmedetomidine. Therefore, genetic variations in CYP2A6 may impact the metabolism of dexmedetomidine and even the drug effect. Populations of different ethnicities exist distinct patterns of *CYP2A6* genetic variation (Tanner and Tyndale, 2017). As a result, CYP2A6 activity is generally lower in Asians than in Europeans (Nakajima et al., 2006).

CYP2A6*2 (479T > A, rs1801272) is a missense mutation that encodes an inactive enzyme with an amino acid substitution of L160H (Carter et al., 2004). It is a rare variant (MAF <0.05) with a mutation rate of 1–3% in European populations and almost no mutations in Asian populations; therefore, it was not included in the study. The whole gene deletion allele CYP2A6*4 will alter the TATA box, which results in a lower expression mRNA of metabolic enzymes (Loukola et al., 2015). There was no statistical association between CYP2A6*4 and dexmedetomidine pharmacokinetic parameters found in Kohli et al.’s study of 43 patients sedated by dexmedetomidine infusion in the ICU and Wang et al.’s study of 31 patients treated with preoperative dexmedetomidine infusion Kohli et al. (2012); Wang et al. (2018).

In our study, the dexmedetomidine plasma concentration of the 99 participants was measured to calculate pharmacokinetic parameters by non-compartmental analyses. The result indicated that *CYP2A6* rs28399433 correlates with the pharmacokinetics parameters of dexmedetomidine (Cmax and AUC). Studies have indicated that *CYP2A6* rs28399433A > C significantly decreases CYP2A6 enzyme activity (Tanner and Tyndale, 2017). Further analysis of the sedative effect in 194 participants suggested that carriers of the *CYP2A6* rs28399433 minor allele (C) are more likely to achieve sedation after dexmedetomidine infusion. We further analyzed the relationship between pharmacokinetic parameters and the sedative effect in the first 99 participants and revealed a significant correlation between Cmax and the sedative effect. Thus, the sedative effect was more effective at higher plasma drug concentrations. Therefore, it can be concluded that *CYP2A6* rs28399433 rare allele (C) carriers have a lower metabolism rate of dexmedetomidine, resulting in higher drug concentrations and leading to more profound sedation.

The UGT superfamily plays an essential role in the phase II metabolism of dexmedetomidine (Yang et al., 2019). Nineteen UGT proteins have been identified in humans, and they are divided into two families, UGT1 and UGT2, and UGT2 is subdivided into two subfamilies UGT2A and UGT2B (Nakamura et al., 2008). James et al. indicated that the *UGT1A4* or *UGT2B10* genotype does not affect the pharmacokinetics of dexmedetomidine in children (James et al., 2021). Similarly, SNPs in the UGT candidate genes screened in our study were not associated with pharmacokinetic parameters or drug effects of dexmedetomidine in adult female patients.

Bradycardia and initial hypertension are common adverse effects of dexmedetomidine (Mason and Lerman, 2011). Our results suggested that *GABRA2* rs279847 gene polymorphism was significantly associated with the decrease in heart rate induced by dexmedetomidine. After dexmedetomidine infusion, peripheral alpha receptors are activated, leading to initial transient hypertension, which may cause a reflex decrease in heart rate (Ebert et al., 2000). Physiologically, variations in blood pressure are often associated with alterations in heart rate, as an increase in blood pressure reflexively induces a decrease in heart rate (Li et al., 2017). Thus, we analyzed the MAP within 20 min and plotted the trend in MAP in patients with different genotypes of *GABRA2* rs279847. The result showed that the gene polymorphism had a weak effect on MAP, thus excluding the possible interference of blood pressure on heart rate. We hypothesized that the bradycardia induced by dexmedetomidine was associated with GABAergic neurotransmission. Therefore, *GABRA2* rs279847 polymorphism might lead to the sympathetic blockade, causing a variation in heart rate decrease.

**FIGURE 2:**
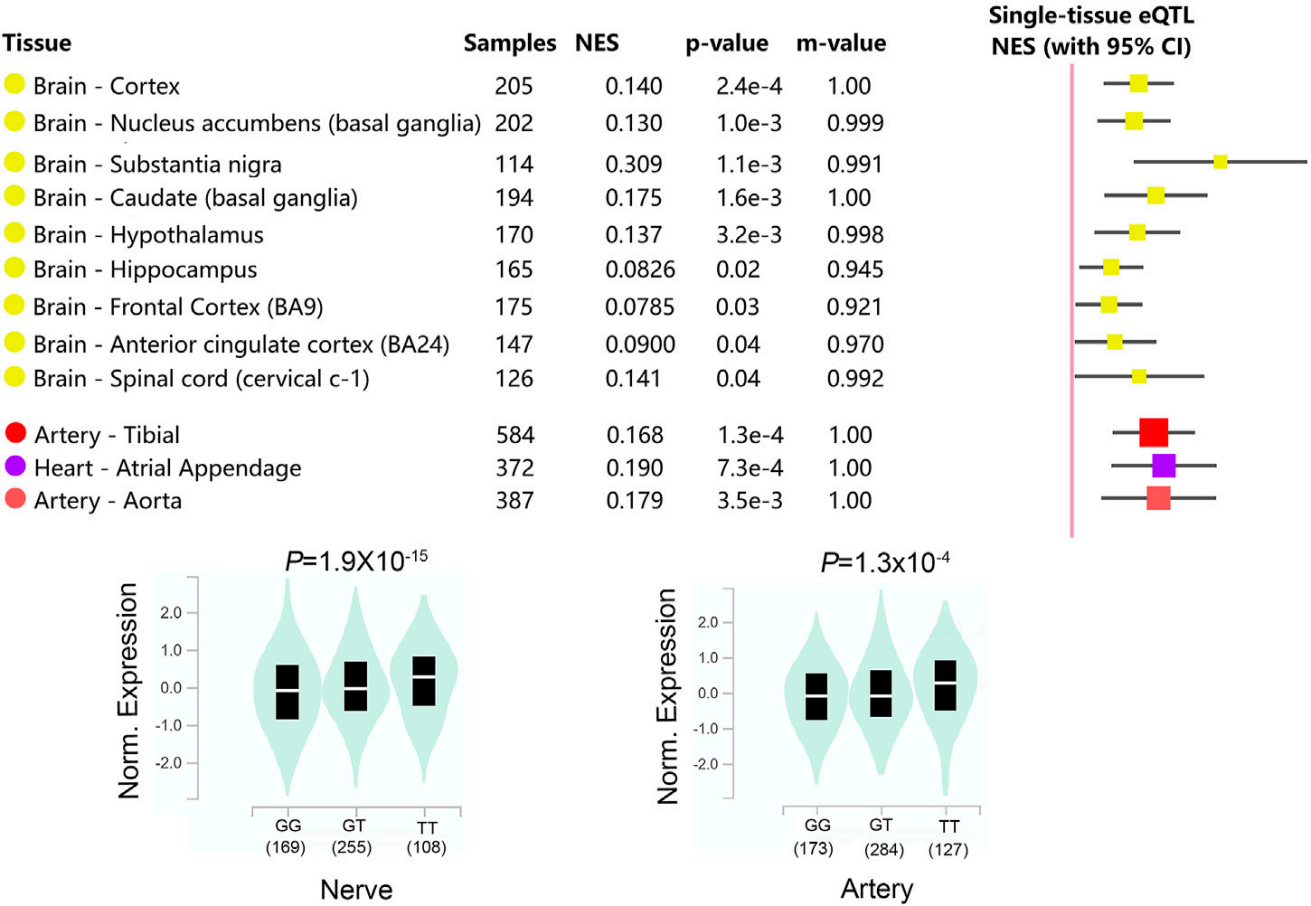
Relationship between *GABRA2* rs279847 and *GABRA2* Expression Quantitative Trait Locus (eQTL) in Multiple Tissues. (from GTEx database, https://www.gtexportal.org/home/).

The hypothalamic paraventricular nucleus is the center of the autonomic system and plays an essential role in regulating cardiovascular activity (Dampney et al., 2018). It has been found that microinjections of GABA into the hypothalamus paraventricular nucleus produced a decrease in heart rate, which was caused by the inhibition of noradrenergic effects in the hypothalamus (Allen, 2002). Based on the analysis of online database, it is found that (Figure 2) the *GABRA2* rs279847 gene polymorphism significantly impacts *GABRA2* expression in the hypothalamus (*p* = 0.0032) and is also significantly associated with *GABRA2* expression in the heart and arterial (*p* < 0.005). This supports our hypothesis that rs279847 alters the sympathetic regulation of GABA by affecting the expression of *GABRA2*, leading to dexmedetomidine-induced heart rate abnormalities. In summary, our results indicated that G carriers of *GABRA2* rs279847 experienced a more significant heart rate decrease after dexmedetomidine infusion.

Overall, decreased *CYP2A6* activity in carriers of the minor allele (C) of *CYP2A6* rs28399433 leads to higher drug concentrations during dexmedetomidine infusion. Therefore, appropriate dose reductions for these patients are warranted. In addition, carriers of the minor allele (G) of *GABRA2* rs279847 have a more severe decrease in heart rate on dexmedetomidine infusion. Therefore more attention and advanced prevention in these patients may be necessary. However, several limitations existed in our study. First, studies have shown that gender contributes to differences in gene expression in specific tissues by affecting the transcription factors (Oliva et al., 2020), and to exclude the effect of gender on drug response, our study included only female patients; therefore, the generalizability of the findings in male patients remains for validation. Besides, certain external environmental and internal endogenous factors can be inducible for *CYP2A6* activity. For example, cigarettes can affect the expression of CYP2A6, resulting in altered metabolism of the relevant drugs (Di et al., 2009). Unfortunately, the indicator was not acquired from the subjects in our study. However, considering that our study population originated from urban women in central China, the prevalence of smoking was extremely low considering cultural and economic reasons, and the vast majority were women of childbearing age. Therefore, this factor should have little effect on our results. Finally, although we detected SNPs with high-frequency mutations in Asians, the present method could not typify all SNPs and, therefore, might have missed some SNPs with low mutations but with potential roles. It also led to our inability to delineate the different metabolic activities of CYP2A6 accurately. In conclusion, future studies require covering larger sample sizes, incorporating more potential confounders, and screening more candidate SNPs to comprehensively analyze the effect of genetic polymorphisms on dexmedetomidine drug response.

## Conclusion

Our study indicated that *CYP2A6* rs28399433 polymorphism affects the metabolic rate of dexmedetomidine and is associated with susceptibility to the sedative effects of dexmedetomidine; *GABRA2* rs279847 polymorphism is significantly associated with the degree of the heart rate decrease. These findings might contribute to the precise dosing of dexmedetomidine in the ICU and perioperative period, although further validation is required in the future.

## Data Availability

The original contributions presented in the study are publicly available. This data can be found here: https://submit.ncbi.nlm.nih.gov/subs/variation_org /accession number: SUB11621926.

## References

[B1] Ahmed S. Zhou Z. Zhou J. Chen S. Q. (2016). Pharmacogenomics of Drug Metabolizing Enzymes and Transporters: Relevance to Precision Medicine. Genomics Proteomics Bioinforma. 14, 298–313. 10.1016/j.gpb.2016.03.008 PMC5093856 27729266

[B2] Allen A. M. (2002). Inhibition of the Hypothalamic Paraventricular Nucleus in Spontaneously Hypertensive Rats Dramatically Reduces Sympathetic Vasomotor Tone. Hypertension 39, 275–280. 10.1161/hy0202.104272 11847197

[B3] Bachtiar M. Ooi B. N. S. Wang J. Jin Y. Tan T. W. Chong S. S. (2019). Towards Precision Medicine: Interrogating the Human Genome to Identify Drug Pathways Associated with Potentially Functional, Population-Differentiated Polymorphisms. Pharmacogenomics J. 19, 516–527. 10.1038/s41397-019-0096-y 31578463 PMC6867962

[B4] Barrett J. C. Fry B. Maller J. Daly M. J. (2005). Haploview: Analysis and Visualization of Ld and Haplotype Maps. Bioinformatics 21, 263–265. 10.1093/bioinformatics/bth457 15297300

[B5] Cacabelos R. Cacabelos N. Carril J. C. (2019). The Role of Pharmacogenomics in Adverse Drug Reactions. Expert Rev. Clin. Pharmacol. 12, 407–442. 10.1080/17512433.2019.1597706 30916581

[B6] Carter B. Long T. Cinciripini P. (2004). A Meta-Analytic Review of the Cyp2a6 Genotype and Smoking Behavior. Nicotine Tob. Res. 6, 221–227. 10.1080/14622200410001676387 15203795

[B7] Chang P. Zhang Y. Gong D. Yang L. Wang J. Liu J. (2020). Determination of Dexmedetomidine Using High Performance Liquid Chromatography Coupled with Tandem Mass Spectrometric (Hplc-Ms/Ms) Assay Combined with Microdialysis Technique: Application to A Pharmacokinetic Study. J. Chromatogr. B Anal. Technol. Biomed. Life Sci. 1160, 122381. 10.1016/j.jchromb.2020.122381 32947190

[B8] Chaudhary R. Singh B. Kumar M. Gakhar S. K. Saini A. K. Parmar V. S. (2015). Role of Single Nucleotide Polymorphisms in Pharmacogenomics and Their Association with Human Diseases. Drug Metab. Rev. 47, 281–290. 10.3109/03602532.2015.1047027 25996670

[B9] Colin P. J. Hannivoort L. N. Eleveld D. J. Reyntjens K. M. E. M. Absalom A. R. Vereecke H. E. M. (2017). Dexmedetomidine Pharmacodynamics in Healthy Volunteers: 2. Haemodynamic Profile. Br. J. Anaesth. 119, 211–220. 10.1093/bja/aex086 28854543

[B10] Correa-Sales C. Rabin B. C. Maze M. (1992). A Hypnotic Response to Dexmedetomidine, an Alpha 2 Agonist, Is Mediated in the Locus Coeruleus in Rats. Anesthesiology 76, 948–952. 10.1097/00000542-199206000-00013 1350889

[B11] Cortínez L. I. Anderson B. J. Holford N. H. Puga V. De La Fuente N. Auad H. (2015). Dexmedetomidine Pharmacokinetics in the Obese. Eur. J. Clin. Pharmacol. 71, 1501–1508. 10.1007/s00228-015-1948-2 26407689

[B12] Dampney R. A. Michelini L. C. Li D. P. Pan H. L. (2018). Regulation of Sympathetic Vasomotor Activity by the Hypothalamic Paraventricular Nucleus in Normotensive and Hypertensive States. Am. J. Physiol. Heart Circ. Physiol. 315, H1200–H1214. 10.1152/ajpheart.00216.2018 30095973 PMC6297824

[B13] Di Y. M. Chow V. D. Yang L. P. Zhou S. F. (2009). Structure, Function, Regulation and Polymorphism of Human Cytochrome P450 2a6. Curr. Drug Metab. 10, 754–780. 10.2174/138920009789895507 19702528

[B14] Ding X. Kaminsky L. S. (2003). Human Extrahepatic Cytochromes P450: Function in Xenobiotic Metabolism and Tissue-Selective Chemical Toxicity in the Respiratory and Gastrointestinal Tracts. Annu. Rev. Pharmacol. Toxicol. 43, 149–173. 10.1146/annurev.pharmtox.43.100901.140251 12171978

[B15] Doyle D. J. Goyal A. Garmon E. H. (2022). American Society of Anesthesiologists Classification. *Statpearls.* Treasure Island (Fl). 28722969

[B16] Ebert T. J. Hall J. E. Barney J. A. Uhrich T. D. Colinco M. D. (2000). The Effects of Increasing Plasma Concentrations of Dexmedetomidine in Humans. Anesthesiology 93, 382–394. 10.1097/00000542-200008000-00016 10910487

[B17] Fatemi S. H. Folsom T. D. Rooney R. J. Thuras P. D. (2013). Expression of Gabaa Alpha2-, Beta1- and Epsilon-Receptors Are Altered Significantly in the Lateral Cerebellum of Subjects with Schizophrenia, Major Depression and Bipolar Disorder. Transl. Psychiatry 3, E303. 10.1038/tp.2013.64 24022508 PMC3784760

[B19] Gertler R. Brown H. C. Mitchell D. H. Silvius E. N. (2001). Dexmedetomidine: A Novel Sedative-Analgesic Agent. Proc. (Bayl Univ. Med. Cent. 14, 13–21. 10.1080/08998280.2001.11927725 16369581 PMC1291306

[B21] James N. T. Breeyear J. H. Caprioli R. Edwards T. Hachey B. Kannankeril P. J. (2021). Population Pharmacokinetic Analysis of Dexmedetomidine in Children Using Real-World Data from Electronic Health Records and Remnant Specimens. Br. J. Clin. Pharmacol. 10.1111/bcp.15194 PMC9106818 34957589

[B22] Kohli U. Pandharipande P. Muszkat M. Sofowora G. G. Friedman E. A. Scheinin M. (2012). Cyp2a6 Genetic Variation and Dexmedetomidine Disposition. Eur. J. Clin. Pharmacol. 68, 937–942. 10.1007/s00228-011-1208-z 22271297 PMC3352974

[B23] Lee S. (2019). Dexmedetomidine: Present and Future Directions. Korean J. Anesthesiol. 72, 323–330. 10.4097/kja.19259 31220910 PMC6676029

[B24] Li L. Wang Y. Uppoor R. S. Mehta M. U. Farchione T. Mathis M. V. (2017). Exposure-Response Analyses of Blood Pressure and Heart Rate Changes for Methylphenidate in Healthy Adults. J. Pharmacokinet. Pharmacodyn. 44, 245–262. 10.1007/s10928-017-9513-5 28214989

[B25] Liu X. Li Y. Kang L. Wang Q. (2021). Recent Advances in the Clinical Value and Potential of Dexmedetomidine. J. Inflamm. Res. 14, 7507–7527. 10.2147/JIR.S346089 35002284 PMC8724687

[B26] Loukola A. Buchwald J. Gupta R. Palviainen T. Hällfors J. Tikkanen E. (2015). A Genome-wide Association Study of A Biomarker of Nicotine Metabolism. Plos Genet. 11, E1005498. 10.1371/journal.pgen.1005498 26407342 PMC4583245

[B27] Mason K. P. Lerman J. (2011). Review Article: Dexmedetomidine in Children: Current Knowledge and Future Applications. Anesth. Analg. 113, 1129–1142. 10.1213/ANE.0b013e31822b8629 21821507

[B28] Mondello E. Siliotti R. Noto G. Cuzzocrea E. Scollo G. Trimarchi G. (2002). Bispectral Index in Icu: Correlation with Ramsay Score on Assessment of Sedation Level. J. Clin. Monit. Comput. 17, 271–277. 10.1023/a:1021250320103 12546259

[B29] Muszkat M. Kurnik D. Solus J. Sofowora G. G. Xie H. G. Jiang L. (2005). Variation in the Alpha2b-Adrenergic Receptor Gene (Adra2b) and its Relationship to Vascular Response *In Vivo* . Pharmacogenet Genomics 15, 407–414. 10.1097/01213011-200506000-00006 15900214

[B30] Nakajima M. Fukami T. Yamanaka H. Higashi E. Sakai H. Yoshida R. (2006). Comprehensive Evaluation of Variability in Nicotine Metabolism and Cyp2a6 Polymorphic Alleles in Four Ethnic Populations. Clin. Pharmacol. Ther. 80, 282–297. 10.1016/j.clpt.2006.05.012 16952495

[B31] Nakamura A. Nakajima M. Yamanaka H. Fujiwara R. Yokoi T. (2008). Expression of Ugt1a and Ugt2b Mrna in Human Normal Tissues and Various Cell Lines. Drug Metab. Dispos. 36, 1461–1464. 10.1124/dmd.108.021428 18480185

[B32] Oliva M. Muñoz-Aguirre M. Kim-Hellmuth S. Wucher V. Gewirtz A. D. H. Cotter D. J. (2020). The Impact of Sex on Gene Expression across Human Tissues. Science 369. 10.1126/science.aba3066 PMC8136152 32913072

[B33] Philbin K. E. Bateman R. J. Mendelowitz D. (2010). Clonidine, an Alpha2-Receptor Agonist, Diminishes Gabaergic Neurotransmission to Cardiac Vagal Neurons in the Nucleus Ambiguus. Brain Res. 1347, 65–70. 10.1016/j.brainres.2010.06.001 20553874 PMC2909326

[B36] Smithburger P. L. Smith R. B. Kane-Gill S. L. Empey P. E. (2014). Patient Predictors of Dexmedetomidine Effectiveness for Sedation in Intensive Care Units. Am. J. Crit. Care 23, 160–165. 10.4037/ajcc2014678 24585165 PMC4132632

[B37] Talke P. Stapelfeldt C. Lobo E. Brown R. Scheinin M. Snapir A. (2005a). Alpha-2b Adrenoceptor Polymorphism and Peripheral Vasoconstriction. Pharmacogenet Genomics 15, 357–363. 10.1097/01213011-200505000-00012 15864138

[B38] Talke P. Stapelfeldt C. Lobo E. Brown R. Scheinin M. Snapir A. (2005b). Effect of Alpha2b-Adrenoceptor Polymorphism on Peripheral Vasoconstriction in Healthy Volunteers. Anesthesiology 102, 536–542. 10.1097/00000542-200503000-00010 15731590

[B39] Tanner J. A. Tyndale R. F. (2017). Variation in Cyp2a6 Activity and Personalized Medicine. J. Pers. Med. 7. 10.3390/jpm7040018 PMC5748630 29194389

[B40] Wang L. Wang S. Qi J. Yu R. Zhuang J. Zhuang B. (2018). Impact of Cyp2a6 Gene Polymorphism on the Pharmacokinetics of Dexmedetomidine for Premedication. Expert Rev. Clin. Pharmacol. 11, 917–922. 10.1080/17512433.2018.1510312 30092666

[B41] Waring R. H. (2020). Cytochrome P450: Genotype to Phenotype. Xenobiotica 50, 9–18. 10.1080/00498254.2019.1648911 31411087

[B42] Yagar S. Yavas S. Karahalil B. (2011). The Role of the Adra2a C1291g Genetic Polymorphism in Response to Dexmedetomidine on Patients Undergoing Coronary Artery Surgery. Mol. Biol. Rep. 38, 3383–3389. 21104443 10.1007/s11033-010-0446-y

[B43] Yang M. Tse A. H. W. Lee A. Joynt G. M. Zuo Z. (2019). Large Inter-individual Variability in Pharmacokinetics of Dexmedetomidine and its Two Major N-Glucuronides in Adult Intensive Care Unit Patients. J. Pharm. Biomed. Anal. 175, 112777. 10.1016/j.jpba.2019.07.025 31362246

[B44] Zhu S. J. Wang K. R. Zhang X. X. Zhu S. M. (2019). Relationship between Genetic Variation in the α2A-adrenergic Receptor and the Cardiovascular Effects of Dexmedetomidine in the Chinese Han Population. J. Zhejiang Univ. Sci. B 20, 598–604. 10.1631/jzus.B1800647 31168973 PMC6586997

