# Supplementary material for: CYP2A6 and GABRA2 Gene Polymorphisms are Associated With Dexmedetomidine Drug Response
Source: Front Pharmacol. 2022 Jul 7;13:943200. doi: 10.3389/fphar.2022.943200 (PMC9301121; doi:10.3389/fphar.2022.943200)
Supplement: Supplementary file 1 [file DataSheet1.docx]

**Supplementary Table S1** The sequences of PCR primers

| rs143731390 | Forward primer | 5’-CGGTGGTGAAGAAGAGAAAG-3’ |
| --- | --- | --- |
|  | Reverse primer | 5’-ACTGAGAGTGGGCTTCACCT-3’ |
| rs28399433 | Forward primer | 5’-TGGGATGATAGATGGTGACG-3’ |
|  | Reverse primer | 5’-TAGGCAGGATTCATGGTGGG-3’ |
| rs28399481 | Forward primer | 5’-AGCACCTTATCAAGGTGAAC-3’ |
|  | Reverse primer | 5’-AGAGGGGCGCAGCTAAGACT-3’ |
| rs5031016 | Forward primer | 5’-TGTAGTTTCGTGGGATCGTG-3’ |
|  | Reverse primer | 5’-TTCTCTTCTTCACCACCGTC-3’ |
| rs115455627 | Forward primer | 5’-AAATGACATTTCACCTAGCC-3’ |
|  | Reverse primer | 5’-TACCCTCCTTACACAAAGGC-3’ |
| rs11940320 | Forward primer | 5’-AGACCTTGCCAGTGTCTAAC-3’ |
|  | Reverse primer | 5’-AGTGAGATTATGGCACTCCC-3’ |
| rs12468356 | Forward primer | 5’-CATCCTGGGGTTTTTGCTTG-3’ |
|  | Reverse primer | 5’-CTGTGGTCACCTCTGTATCT-3’ |
| rs8330 | Forward primer | 5’-CAGATGGTTGCAATTGATCC-3’ |
|  | Reverse primer | 5’-CTAAAGGTACAAAGCTTCCC-3’ |
| rs2008584 | Forward primer | 5’-AGACTGTGCAAGATCTGAGG-3’ |
|  | Reverse primer | 5’-GAAGAAGAGGTGCTGCTATC-3’ |
| rs2484516 | Forward primer | 5’-AGAGCTGATCGTTCACCTGC-3’ |
|  | Reverse primer | 5’-ACTTCCAAAGTTGTGCGCCC-3’ |
| rs3750625 | Forward primer | 5’-TACCTAGCCCTGGCTAATTC-3’ |
|  | Reverse primer | 5’-TAGTGTATATTTACAGCGGG-3’ |
| rs4907299 | Forward primer | 5’-GGGTGATCAGTCTTCGTTTC-3’ |
|  | Reverse primer | 5’-GCTTGTGGTGTTTTCGTTTC-3’ |
| rs2229169 | Forward primer | 5’-AGTAGCCGATCCAGAAGAAG-3’ |
|  | Reverse primer | 5’-TTTTGTGCTCTGCTGGTTCC-3’ |
| rs7434444 | Forward primer | 5’-GTCAGGGTTTTAGAGAGCAG-3’ |
|  | Reverse primer | 5’-AGAAGTCAGCCCTTGGTCAG-3’ |
| rs11269124 | Forward primer | 5’-TGGGAATCCTGGACAGCTC-3’ |
|  | Reverse primer | 5’-CCCTGGAGGCCAATCCATC-3’ |
| rs11576001 | Forward primer | 5’-CTGATGTTGAAACACACCCC-3’ |
|  | Reverse primer | 5’-AGAAGTCAGGACGAAATCCG-3’ |
| rs77445936 | Forward primer | 5’-CAATATATAGGACAGCCACC-3’ |
|  | Reverse primer | 5’-GAGTGATCTTTCACATGTGC-3’ |
| rs279847 | Forward primer | 5’-GATCAGTCTTCTCTATCCCC-3’ |
|  | Reverse primer | 5’-GCTTTTTAGATGCCGGTGAC-3’ |
| rs10214094 | Forward primer | 5’-GGAACTCAAAGGACATACAG-3’ |
|  | Reverse primer | 5’-GCAGCACTCTTAAAAAGCTAC-3’ |
| rs10433685 | Forward primer | 5’-CCCTGATAAGTACAATTGGC-3’ |
|  | Reverse primer | 5’-CCAATTGCTCCCAGACATAG-3’ |
| rs4076138 | Forward primer | 5’-TGGCCTTTGGATTCTGAGTC-3’ |
|  | Reverse primer | 5’-GTTCAGGTCCACTAGAACAC-3’ |
| rs4958283 | Forward primer | 5’-GGCTCTCAAACTAGTAGAGG-3’ |
|  | Reverse primer | 5’-TTTCCACTCTGAGATTGCAC-3’ |

**Supplementary Table S2** Effects of the remaining six metabolic enzyme gene SNPs on pharmacokinetic parameters of dexmedetomidine

| **Polymorphism** | **Genotype** | **n** | **HL_Lambda_z (h)** | **Cmax**  **(μg/L)** | **AUClast (h*μg/L)** | **AUCINF_obs(h*μg/L)** | **Vz_obs (L/kg)** | **Cl_obs (L/h/kg)** |
| --- | --- | --- | --- | --- | --- | --- | --- | --- |
| *UGT2B10* | | | | | | | | |
| rs115455627 | CC | 61 | 2.62±0.85 | 2.14±0.73 | 1.45±0.45 | 1.64±0.52 | 2.27±0.58 | 0.65±0.25 |
|  | CT | 28 | 2.59±1.89 | 1.97±0.82 | 1.46±0.66 | 1.76±0.52 | 1.97±0.74 | 0.61±0.25 |
|  | TT | 8 | 3.66±3.87 | 1.87±0.66 | 1.35±0.67 | 1.90±1.78 | 2.05±0.92 | 0.82±0.48 |
| rs11940320 | GG | 79 | 2.68±1.73 | 2.00±0.72 | 1.43±0.54 | 1.67±0.83 | 2.14±0.70 | 0.65±0.29 |
|  | AG+AA | 19+1 | 2.87±1.03 | 2.27±0.84 | 1.51±0.47 | 1.76±0.61 | 2.38±0.51 | 0.63±0.21 |
| *UGT1A4* | | | | | | | | |
| rs12468356 | AA | 67 | 2.77±1.85 | 2.12±0.73 | 1.45±0.52 | 1.72±0.85 | 2.19±0.68 | 0.66±0.29 |
|  | GA+GG | 30+2 | 2.61±0.94 | 1.93±0.77 | 1.44±0.55 | 1.63±0.64 | 2.19±0.66 | 0.64±0.24 |
| rs2008584 | GG | 49 | 2.63±1.75 | 2.08±0.69 | 1.44±0.56 | 1.68±0.87 | 2.10±0.71 | 0.67±0.32 |
|  | GA | 48 | 2.64±0.89 | 2.04±0.82 | 1.45±0.49 | 1.65±0.56 | 2.23±0.62 | 0.63±0.21 |
| rs8830 | CC | 81 | 2.74±1.73 | 2.01±0.73 | 1.41±0.49 | 1.67±0.82 | 2.24±0.62 | 0.65±0.26 |
|  | GC+GG | 17+1 | 2.60±0.96 | 2.27±0.82 | 1.61±0.65 | 1.78±0.67 | 1.96±0.86 | 0.67±0.34 |
| *CYP2A6* | | | | | | | | |
| rs143731390 | TT | 76 | 2.78±1.77 | 2.13±0.78 | 1.47±0.54 | 1.74±0.85 | 2.13±0.68 | 0.62±0.25 |
|  | TA | 21 | 2.44±0.92 | 1.83±0.64 | 1.34±0.50 | 1.55±0.56 | 2.34±0.64 | 0.74±0.34 |

**Supplementary Table S3** SNPs with no statistically significant influence on the sedative effect of dexmedetomidine

| **Gene** | **SNPs** | **Genotype** | **Ramsay≥4** | | **OR (95%CI)** | ***P* value** |
| --- | --- | --- | --- | --- | --- | --- |
|  |  |  | **Y** | **N** |  |  |
| *UGT2B10* | rs11940320 | GG | 140 | 20 | 1.40 (0.54-3.64) | 0.49 |
|  |  | AG+AA | 27+1 | 6 |  |  |
|  | rs115455627 | CC | 110 | 19 | 0.77 (0.36-1.65) | 0.50 |
|  |  | CT | 49 | 4 |  |  |
|  |  | TT | 9 | 2 |  |  |
| *GABRA2* | rs279847 | GG | 42 | 6 | 1.05 (0.60-1.83) | 0.86 |
|  |  | GT | 73 | 13 |  |  |
|  |  | TT | 53 | 7 |  |  |
|  | rs10433685 | GG | 62 | 8 | 0.88 (0.45-1.70) | 0.70 |
|  |  | CG | 106 | 18 |  |  |
| *UGT1A4* | rs12468356 | AA | 103 | 20 | 0.58(0.24-1.36) | 0.21 |
|  |  | GA | 60 | 5 |  |  |
|  |  | GG | 5 | 1 |  |  |
|  | rs8330 | CC | 135 | 18 | 1.74 (0.72-4.19) | 0.21 |
|  |  | GC | 33 | 7 |  |  |
| *GLRA1* | rs4076138 | CC | 13 | 2 | 1.15(0.60-2.18) | 0.68 |
|  |  | CT | 68 | 12 |  |  |
|  |  | TT | 87 | 12 |  |  |
|  | rs4958283 | AA | 65 | 9 | 1.24 (0.70-2.20) | 0.46 |
|  |  | AG | 76 | 11 |  |  |
|  |  | GG | 27 | 6 |  |  |
| *GABRB2* | rs10214094 | AA | 140 | 22 | 0.90 (0.29-2.82) | 0.86 |
|  |  | GA | 28 | 4 |  |  |
| *ADRA2A* | rs2484516 | CC | 137 | 22 | 0.64 (0.18-2.20) | 0.47 |
|  |  | CG+GG | 28+1 | 3 |  |  |
| *CYP2A6* | rs143731390 | AT | 40 | 10 | 1.97 (0.83-4.68) | 0.13 |
|  |  | TT | 126 | 16 |  |  |
| *ADRA2B* | rs4907299 | GG | 41 | 4 | 0.80 (0.45-1.44) | 0.13 |
|  |  | GT | 79 | 14 |  |  |
|  |  | TT | 48 | 8 |  |  |
|  | rs2229169 | GG | 52 | 6 | 1.11 (0.62-1.97) | 0.74 |
|  |  | GT | 79 | 15 |  |  |
|  |  | TT | 37 | 5 |  |  |
| *ADRA2C* | rs11269124 | GG | 16 | 1 | 0.55 (0.27-1.13) | 0.10 |
|  |  | DEL.G | 82 | 10 |  |  |
|  |  | DEL. | 70 | 15 |  |  |
| *GABRA1* | rs77445936 | TT | 108 | 14 | 1.56 (0.79-3.12) | 0.20 |
|  |  | CT | 55 | 10 |  |  |
|  |  | CC | 5 | 2 |  |  |
|  | rs11576001 | AA | 48 | 6 | 1.46 (0.80-2.67) | 1.22 |
|  |  | GA | 89 | 12 |  |  |
|  |  | GG | 31 | 8 |  |  |

**Supplementary Table S4** SNPs with no statistically significant influence on the abnormal heart rate due to dexmedetomidine

| **Genes** | **SNPs** | **Genotype** | **HR<50** | | **OR (95%CI)** | **P value** |
| --- | --- | --- | --- | --- | --- | --- |
|  |  |  | **N** | **Y** |  |  |
| *UGT2B10* | rs11940320 | GG | 129 | 31 | 1.02 (0.42-2.48) | 0.96 |
|  |  | GA+AA | 26+1 | 7 |  |  |
|  | rs115455627 | CC | 99 | 27 | 0.60 (0.30-1.21) | 0.16 |
|  |  | CT | 43 | 10 |  |  |
|  |  | TT | 11 | 0 |  |  |
| *UGT1A4* | rs12468356 | AA | 97 | 27 | 0.62 (0.30-1.27) | 0.19 |
|  |  | AG | 53 | 11 |  |  |
|  |  | GG | 6 | 0 |  |  |
|  | rs8330 | CC | 126 | 28 | 1.40 (0.63-3.09) | 0.41 |
|  |  | CG | 29 | 10 |  |  |
|  |  | GG | 1 | 0 |  |  |
| *CYP2A6* | rs143731390 | AT | 28 | 22 | 1.01 (0.53-1.94) | 0.96 |
|  |  | TT | 79 | 63 |  |  |
|  | rs28399433 | AA | 85 | 15 | 1.81 (1.07-3.04) | 0.026 |
|  |  | AC | 59 | 16 |  |  |
|  |  | CC | 11 | 7 |  |  |
| *GLRA1* | rs4958283 | GG | 60 | 14 | 1.00 (0.61-1.65) | 0.99 |
|  |  | GA | 68 | 18 |  |  |
|  |  | AA | 28 | 0 |  |  |
|  | rs4076138 | TT | 80 | 19 | 1.13 (0.65-1.97) | 0.66 |
|  |  | TC | 65 | 15 |  |  |
|  |  | CC | 11 | 4 |  |  |
| *ADRA2A* | rs2484516 | CC | 129 | 30 | 1.13 (0.46-2.75) | 0.79 |
|  |  | CG+GG | 24+1 | 7 |  |  |
| *ADRA2B* | rs2229169 | GG | 48 | 10 | 0.99 (0.60-1.64) | 0.99 |
|  |  | GT | 73 | 21 |  |  |
|  |  | GG | 35 | 7 |  |  |
|  | rs4907299 | TT | 45 | 11 | 0.90 (0.55-1.48) | 0.68 |
|  |  | TG | 74 | 20 |  |  |
|  |  | GG | 37 | 7 |  |  |
| *ADRA2C* | rs11269124 | DEL. | 65 | 18 | 1.00 (0.57-1.76) | 0.98 |
|  |  | DEL.G | 78 | 15 |  |  |
|  |  | GG | 12 | 5 |  |  |
| *GABRA1* | rs77445936 | TT | 97 | 26 | 0.86 (0.45-1.66) | 0.66 |
|  |  | TC | 54 | 10 |  |  |
|  |  | CC | 5 | 2 |  |  |
|  | rs11576001 | AA | 44 | 11 | 0.8 (0.52-1.46) | 0.59 |
|  |  | AG | 79 | 21 |  |  |
|  |  | GG | 33 | 6 |  |  |
| *GABRA2* | rs10433685 | GG | 59 | 12 | 1.26 (0.72-2.20) | 0.42 |
|  |  | GC | 82 | 21 |  |  |
|  |  | CC | 15 | 5 |  |  |
| *GABRB2* | rs10214094 | AA | 127 | 33 | 0.71 (0.25-1.99) | 0.52 |
|  |  | AG | 28 | 5 |  |  |
